# Defective Calculations

**Published:** 1883-04

**Authors:** 


Defective Calculations.—Laying aside these generalities, let us consider an example of the way in which we can weigh and measure, submit the results to calculation, and draw from them conclusions which are formally quite legitimate, and still be all the time on the wrong track; then examine how we may be set upon the right road, and lead to a new conclusion more plausible and more in harmony with the rest of our knowledge.

It has been discovered that the flea can leap two hundred times its own length. Our admiration at this is changed to astonishment when it is demonstratad by calculation that, if nature had endowed the horse with a degree of strength proportioned to his weight he would have been able to clear the Rocky Mountains at a bound, and that with a like effort a whale would be able to leap to a height of two hundred leagues. What can be more unassailable than these conclusions, founded on weight, measure, and calculation?

It is true that if, instead of comparing the weight of the horse and the flea, we had compared their heights, we should have found that the horse's leap would not measure more than three hundred metres. Why is preference given to the weight? Because it is its whole body, with its three dimensions and its density, that the flea hurls to two hundred times its height, and it is the same feat of strength that we demand in vain of the horse. Calculations have also been made to show that if a man could move with a speed proportioned to that of certain insects, he would be able to travel more than ten leagues in a minute, or sixty times as fast as a railroad train.

The Amazon ants, going to battle, travel from two to two and a half metres a minute. The Amazons of antiquity, to be even with them, if we judge by the relative heights, should have traveled eight leagues an hour. We have, however, in this case, to compare the forces with which given masses move themselves, and take account of weights or volumes. If we proceed by this rule we shall obtain formidable numbers that stagger the boldest imagination. The warlike inhabitants of the banks of the Thermodon would have to get over fifty thousand leagues in an hour. Yet who can deny the truth of the observations, the rigor of the measurements, or the justice of the reasoning?—*From "Dwarfs and Giants," by* M. Delbœuf, *in Pop. Science Monthly for April.*

